# Weekly oxaliplatin, 5-fluorouracil and folinic acid (OXALF) as first-line chemotherapy for elderly patients with advanced gastric cancer: results of a phase II trial

**DOI:** 10.1186/1471-2407-6-125

**Published:** 2006-05-10

**Authors:** D Santini, F Graziano, V Catalano, M Di Seri, E Testa, AM Baldelli, P Giordani, A La Cesa, B Spalletta, B Vincenzi, A Russo, M Caraglia, V Virzi, S Cascinu, G Tonini

**Affiliations:** 1 Medical Oncology, University Campus Bio-Medico, Rome, Italy; 2 Medical Oncology, Civic Hospital, Urbino, Italy; 3 Medical Oncology, Civic Hospital, Pesaro, Italy; 4 Medical Oncology, University La Sapienza, Rome, Italy; 5 Medical Oncology, University of Palermo, Italy; 6 National Cancer Institute Fondazione "G. Pascale", Experimental Oncology, Department, Experimental Pharmacology Unit, Naples, Italy; 7 Division of Medical Oncology, University of Ancona, Ancona, Italy

## Abstract

**Background:**

Elderly patients have been often excluded from or underrepresented in the study populations of combination chemotherapy trials. The primary end point of this study was to determine the response rate and the toxicity of the weekly oxaliplatin, 5-fluorouracil and folinic acid (OXALF) regimen in elderly patients with advanced gastric cancer. The secondary objective was to measure the time to disease progression and the survival time.

**Methods:**

Chemotherapy-naive patients with advanced gastric cancer aged 70 or older were considered eligible for study entry. Patients received weekly oxaliplatin 40 mg/m2, fluorouracil 500 mg/m2 and folinic acid 250 mg/m2. All drugs were given intravenously on a day-1 schedule.

**Results:**

A total of 42 elderly patients were enrolled. Median age was 73 years and all patients had metastatic disease. The response rate according to RECIST criteria was 45.2% (95% CIs: 30%–56%) with two complete responses, 17 partial responses, 13 stable diseases and 10 progressions, for an overall tumor rate control of 76.2% (32 patients). Toxicity was generally mild and only three patients discontinued treatment because of treatment related adverse events. The most common treatment-related grade 3/4 adverse events were fatigue (7.1%), diarrhoea (4.8%), mucositis (2.4%), neurotoxicity (2.4%) and neutropenia (4.8%). The median response duration was 5.3 months (95% CIs: 2.13 – 7.34), the median time to disease progression was 5.0 months (95% CIs: 3.75 – 6.25) and the median survival time was 9.0 months (95% CIs: 6.18 – 11.82).

**Conclusion:**

OXALF represents an active and well-tolerated treatment modality for elderly patients with locally advanced and metastatic gastric cancer.

## Background

Gastric cancer is the fourth most frequent malignant disease and it is among the most common causes of cancer-related death. Current epidemiological data indicate that gastric cancer rarely occurs before the age of 40 years, its incidence increases thereafter and peaks in the seventh decade. Patients older than 65 years have been often excluded from or underrepresented in the study populations of combination chemotherapy trials [1]. This demographic selection occurs in populations of gastric cancer patients treated with combination chemotherapy regimens. Demographic analysis of phase II and III chemotherapy trials show that only a minority of treated patients were older than 65 years [2,3]. Consequently, there is uncertainty about the type and the extent of systemic palliative chemotherapy that should be offered to elderly patients. In 2003, Graziano et al. [4] showed that the weekly PLF (cisplatin/leucovorin/fluorouracil) chemotherapy may represent a valid and safety alternative for elderly patients with advanced gastric cancer.

Oxaliplatin is an alkylating agent inhibiting DNA replication by forming adducts between two adjacent guanines or guanine and adenine molecules. The adducts of oxaliplatin appear to be more effective than cisplatin adducts with regard to the inhibition of DNA synthesis [5]. Many studies are ongoing to test the oxaliplatin-based combinations in non-colorectal gastrointestinal carcinomas and other malignancies. Few studies have been addressed to the analysis of oxaliplatin-based chemotherapy in advanced gastric cancer [6-11]. The oxaliplatin dose-limiting toxicity is a cumulative sensory peripheral neuropathy. However, oxaliplatin may offer a more favourable toxicity profile and higher activity compared to cisplatin.

We investigated in the present phase II study a weekly protocol with oxaliplatin, fluorouracil and folinic acid for treating elderly patients with advanced gastric cancer.

## Methods

### Patients characteristics

Chemotherapy-naive pts with advanced gastric cancer [at least one measurable lesion] aged 70 or older were considered eligible for study entry. Inclusion criteria consisted of: ECOG performance status 0 or I, normal renal, liver, bone marrow functions and measurable disease. The Katz and Lawton scales were used to assess activities of daily living [12]; the Katz activities of daily living (ADL) measures the ability to perform routine activities as bathing, dressing, feeding oneself or getting into or out of bed, chairs and vehicles. The Lawton instrumental activities of daily living (IADL) measures more particular functions as the ability to use the telephone, to shop, to handle money, to prepare food or to perform other household tasks. As an adjunct to general and cancer-specific diagnostic procedures, the comprehensive geriatric assessment (CGA) was used at the study entry to identify and exclude frail elderly patients. Frail elderly pts were excluded after baseline geriatric assessment according to the following criteria: age > 85 years, dependence in one or more activities of daily living, presence of three or more comorbidity conditions and presence of one or more geriatric syndromes. Classic geriatric syndromes which had to be excluded before study inclusion were: dementia, delirium, severe depression, frequent falls, neglect and/or abuse, and spontaneous fractures [13]. Life expectancy ≥3 months, no concurrent uncontrolled medical illness, no other malignancies. Patients were excluded if they had National Cancer Institute common toxicity criteria grade ≥2 peripheral neuropathy. The protocol was approved by each local institutional review board and written informed consent was obtained from all participants.

### Study design and dose modifications

Patients received weekly oxaliplatin 40 mg/m2 (90 minutes infusion), fluorouracil 500 mg/m2 (10 minutes bolus) and folinic acid 250 mg/m2 (30 minutes infusion) [OXALF]. All drugs were given intravenously on a day-1 schedule. Chemotherapy was given in an outpatient setting. Antiemetic prophylaxis was given according to local protocols. Most of patients received a pre-medication with 10 mg of metoclopramide without any post-medication. According to treatment policy at each participating institution, the use of growth factors for white cells (G-CSF) and eritropietin was allowed in the case of acute toxicity.

Full doses of the anticancer drug were given if granulocyte count was >1500 microL and platelet count was >100000 microL. In the case of any grade 2 (**G2) **or more toxicity except alopecia, chemotherapy was delayed a week and then restarted after full recovery. Reduction of 25% in all drugs dosing was applied for G3 non-hematological toxicity or for G4 hematologic toxicity in the previous cycle. Patients with unsolved grade 2 or more toxicity after two consecutive treatment delays, or experiencing grade 4 non-hematological toxicity except alopecia went off study.

If peripheral neuropathy persisted between two following cycles, the oxaliplatin dose in the next cycle had to reduced by 50%. If pain was associated with peripheral neuropathy 25% reduction in oxaliplatin dosing was applied. If pain persisted between two following cycles, oxaliplatin dose in the next cycle had to be reduced by 40% Treatment withdrawal was planned in the case of persistent neuropathy with pain (G3) after the dose reduction.

### Statistical plan

The primary end point of this study was to determine the response rate and the toxicity of the weekly OXALF regimen in elderly patients with advanced gastric cancer. The secondary objective was to measure the time to disease progression and the survival time. The expected number of patients for this study was calculated according to a Simon optimal two-stage design. An interim analysis was carried out when the first 18 assessable patients had been recruited. If more than six responses [33.3%] were observed, 24 additional patients were to be recruited; otherwise, the study was to be terminated. The regimen was considered sufficiently active to be submitted for further evaluation if the response rate exceeded 30%. The time to disease progression (TTP) was measured from the date of registration to the date of documented disease progression or death. The survival time was measured from the time of registration to the date of death resulting from any cause.

### Response and toxicity assessment

Responses were classified according to RECIST criteria [14]. Computed tomography (CT) scans of measurable lesions were carried out within 4 weeks before the start of the treatment and at every disease restaging. Responses were to be confirmed by subsequent CT scans 4 to 6 weeks after the initial response documentation. Patients were considered assessable for response if they had early disease progression or had received at least 8 cycles of treatment with at least one tumor assessment.

Patients with responsive or stable disease received six additional weekly cycles and they underwent a second measurement at the end of the treatment program. Follow-up controls were performed every 2 months thereafter. All patients had physical examination and blood chemistries before each weekly administration of chemotherapy and toxicity was graded according to National Cancer Institute common toxicity criteria.

## Results

### Response and survival

From October 2002 to April 2005, 42 elderly patients entered on this study (median age, 73 years; range, 70–81). Three patients did not complete eight weekly cycles due to early progression (two patients), one patients refused chemotherapy after grade III fatigue. Toxicities reported in these three patients were included in the overall analysis of side-effects and they were considered as progressions in the intention-to-treat analysis of response. The baseline clinico-pathologic characteristics of the 42 patients are reported in Table 1. At the end of the treatment program, the best intention-to-treat overall response rate in the 42 patients was 45.2% (95% CIs: 30–56%) with two complete responses (4.7%), 17 partial responses (40.5%), 13 stable diseases (31.0%) and 10 progressions (23.8%), for an overall tumor **control **rate (responses plus stabilizations) of 76.2% (32 patients) (Table 2).

The complete responses were observed in patients with locoregional disease plus lymphnodal metastases and in patients with liver metastases. Fifteen partial responses were observed after eight weekly cycles and two other patients with initial stable disease showed partial response after 14 cycles.

**Figure 1:**
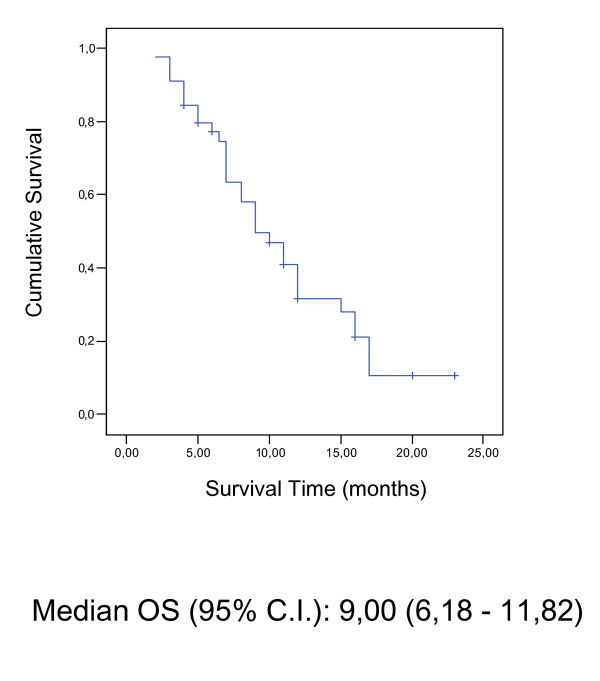


**Figure 2:**
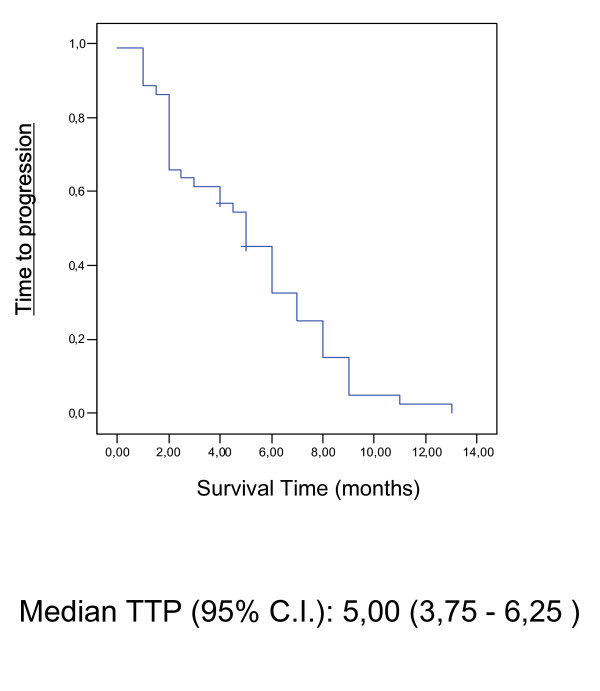


In the whole group, the median follow-up was 14 months [range 3.0–36 months]. The median response duration was 5.3 months (95% CIs: 2,13 – 7,34), the median time to disease progression was 5.0 months (95% CIs: 3,75 – 6,25) and the median survival time was 9.0 months (95% CIs: 6,18 – 11,82) (Table 3). Time To Progression (TTP) and Overall Survival (OS) were assessed by Kaplan-Meier-Analysis shown in Figure 1 and 2.

### Safety

42 patients received a total of 540 treatment cycles. The median number of cycles administered was 12 (range, 3 to 24 cycles). The median cumulative doses in each patient were 480 mg/m^2 ^(range, 80 to 960 mg/m^2^) for oxaliplatin, 6,350 mg/m^2 ^(range, 1,150 to 12,200 mg/m^2^) for 5-FU, and 1,500 mg/m^2 ^(range 250 to 3,000 mg/m^2^) for folinic acid. Toxicity was generally mild and every grade of haematologic and nonhaematologic toxicities per patient is reported in Table 3. 42 patients were included in the safety analysis. Toxicity was generally mild and only three patients discontinued treatment because of treatment related adverse events. Two refused for persistent grade 3 fatigue and the third discontinued for persistent grade 3 neurotoxicity. Overall, 3 patients (7.1%) experienced grade 3 fatigue, 1 patient (2.4%) showed grade 3 neurotoxicity, 2 patients (4.8%) grade 3 neutropenia and another (2.4%) grade 3 mucositis resolved after 1 week delay. Two patients (4.8%) experienced one episode of grade 3 diarrhea resolved after dose reduction. No other grade 3–4 toxicities including peripheral neuropathy were reported and no toxic deaths occurred. No patients received G-CSF with both primary or secondary prophylactic aim. Only two patients received G-CGF for persistent grade 3 neutropenia. These two patients received G-CSF only once for each. Grade 2 nausea and fatigue resulted the commonest non-hematologic toxicity (both, 26.2% of patients). Other grade 2 non-hematologic toxicities were: diarrhea (16.6%), mucositis (11.9%), hyperbilirubinemia (7.1%) and neurotoxicity (11.9%). Two patients (4.8%) reported grade 2 thrombocytopenia, no patient reported grade 2 anemia. Overall, adverse events were reversible and manageable with dose reduction, dose delay or treatment interruption and with symptomatic treatment. Twenty four (57.2%) patients received full doses of all the drugs throughout the study. 5-fluorouracil dose was reduced in 4 patients (9.5%), oxaliplatin dose was reduced in 3 patients (7.1%), and the dose of both agents was reduced in 11 patients (26.2%). The majority of dose reductions were by one level (reduction to 75% of starting dose of fluorouracil and/or of oxaliplatin). Only three patients (7.1%) required a second dose reduction to 50% of starting dose of 5-fluorouracil and/or of oxaliplatin. Adverse events most commonly leading to dose reduction were fatigue and neurotoxicity. The incidence of dose reductions was similar in the subgroups of male and female patients. Only three patients (7.1%) reported a toxicity-related withdrawal with median time to toxicity-related withdrawal of 3.9 months (range: 1.8 – 6 months). The median time to reduction was 2 months (range: 1.0 – 5.3) and the median time to first delay was 1.3 months (range: 0.5 – 3) (Table 4).

The ADL and IADL scores showed non-significant decrases after 8, 12 and 18 weeks of chemotherapy in comparison to baseline scores.

## Discussion

Gastric carcinoma frequently occurs in the 6th and 7th decades and palliative chemotherapy is the ultimate treatment in the majority of patients. The elderly population is expanding and, from the early 1990s, one-quarter of newly diagnosed gastric cancer patients are over 80 years of age [15], but unfortunately, large trials of palliative chemotherapy in elderly patients are almost lacking. To the best of our knowledge, six phase II studies have been published so far [16-21]. In five of these trials, chemotherapy consisted of leucovorin-modulated 5-fluorouracil in combination with cisplatin, epi-doxorubicin, etoposide or mitomycin-C. A phase II study investigated the toxicity profile and the activity of single-agent doxifluridine [21]. In general, the tumour control rate reported was promising and no toxic death was observed across all studies. Unfortunately, these trials often enrolled patients with concomitant illnesses, poor performance status and short life expectancy and consequently, the results in term of survival and safety were heterogeneous and, often, deluding. More recently, Graziano et al [4] have reported the results of a phase II trial investigating the weekly combination of cisplatin, 5-fluorouracil and folinic acid (PLF) in a population advanced gastric cancer patients who were older than 65 year. The regimen showed promising tumour control rate and it was delivered to non-frail elderly patients on outpatient basis. The non-haematological toxicity profile was moderate and haematologic toxicity was comparable to that usually observed in cisplatin-based regimens. Filgrastim support was necessary in several patients and it was maintained until the end of the treatment program. Anaemia was recorded in 31 patients whose haemoglobin concentrations declined to 10–12 g/dl in 26 patients and to 8–9.9 g/dl in 15 patients.

Oxaliplatin may represent an intriguing alternative to cisplatin with at least comparable activity, but a more favourable global toxicity profile. So far, weekly and biweekly FU/FA/oxaliplatin regimens have been mainly explored in colorectal cancer patients with encouraging efficacy data. More recently, weekly [10] and biweekly oxaliplatin-based combinations have been also evaluated in phase II studies of first- [6,7,9] and second- [22] line chemotherapy for patients with advanced gastric cancer. Based on these results, we planned a phase II study to explore safety and efficacy of a weekly FU/FA/oxaliplatin regimen in elderly (> 70 years old) advanced gastric cancer patients. In the interesting Lordick's study [10] the authors chose to use oxaliplatin at the dose of 50 mg/m2 given on days 1, 8, 15 and 22 of a 5-week cycle, while in our study we decided to administer oxaliplatin at the lower dose of 40 mg/m2 every week without any interruption. The dose-intensity of oxaliplatin in our study results to be the same when compared with that calculated in the lordick's study. Moreover, we chose to use a bolus 5-FU/FA regimen because it was more simply to administer, mostly in our elderly setting of patients, when compared with the weekly 24-h infusional regimen. To the best of our knowledge this is the first evaluation of this schedule and it showed promising results. The overall response rate in 42 assessable patients treated with OXALF regimen was 45.2%, including two CRs (4.7%) and 17 PRs (40.5%), and it is comparable to the activity of other chemotherapeutic regimens like FAMTX, ECF, ELF, and FOLFOX6 [25,26,22] and the weekly PLF schedule in elderly patients [4]. Also, the median 5.0 months TTP and the median 9.0 months OS are similar to those reported after ECF, ELF, CF, FAMTX, weekly PLF regimens [25,26]. As far toxicity is concerned, the weekly OXALF regimen seems to cause less haematological toxicity than the weekly PLF regimen, and reduced need for haematopoietic growth factors. In general, higher rates of neutropenia and leucopenia were reported using other cisplatin and oxaliplatin-based protocols in advanced gastric cancer patients (38%, 29% and 36% WHO grade 3/4 neutropenia in ECF, FOLFOX 6 and FOLFOX 4 regimens, respectively). [22-24]. The low incidence of grade 3 toxicity and the absence of any grade 4 toxicity reported in the present study is very important for the setting of elderly patients when compared with the reported incidence of grade 3/4 toxicity in the above mentioned studies [11,22-24]. Moreover, the percent of patients with dose-reduction of 5-FU alone, or oxaliplatin alone or both was 26% (one patient every four) and most of these dose reductions occurred late during the therapy (Table 4), mostly after the first 8 cycles of the regimen. This led us to give some toxicity only to responder-patients avoiding to induce toxicity in non-responder patients (equal or less than 8 cycles).

## Conclusion

In conclusion, the weekly OXALF regimen seems a well-tolerated treatment modality for elderly patients with locally advanced and metastatic gastric cancer. Its promising activity deserves further evaluation in randomized multicenter trials.

## Competing interests

The author(s) declare that they have no competing interests.

## Authors' contributions

DS conceived of the study, participated in its design and coordination and had an important role in the preparation of the manuscript. FG, VC and BV participated in the design of the study and in the conduction of the study. MD, ET, GC, AL, SG, BS, AR, MC, VV, SC and GT participated in the data base compilation and in the conduction of the study. GT supervised all the steps of the study conduction. All authors read and approved the final manuscript.

## Pre-publication history

The pre-publication history for this paper can be accessed here:



## Figures and Tables

**Table 1:** Patient characteristics

***Characteristics***	***No. of patients (%)***
Total number	42 (100%)
Male/Female	26/16 (61.9/38.1)
Age (years)	
median	73
range	70–81
Performance Status	
ECOG 0	18 (42.8)
ECOG 1	24 (57.2)
	
Tumor differentiation	
Well differentiated	10 (23.8)
Moderately differentiated	13 (30.9)
Poorly differentiated or undifferentiated	19 (45.3)
	
Locally advanced disease	10 (23.8)
Metastatic disease	32 (76.2)
Number of metastases	
1	16 (38.2)
2	19 (45.2)
3+	7 (16.6)
Sites of metastases	
Liver	20 (47.6)
Peritoneum	9 (21.4)
Nodes	22 (52.4)
Local	15 (35.7)
Other	6 (14.3)
Prior adjuvant chemotherapy	
None	35 (83.3)
Yes	7 (16.7)
Patients with metastases at the diagnosis	18 (42.8)
Number of cycles	
Total	540
Median	12
Range	3–24

**Table 2:** Objective tumor response rates in advanced untreated elderly gastric cancer patients

***Objective responses***	***Number (%)***	***Overall response rate***	***Overall tumor control rate***
Complete Response	2 (4.7%)	19 (45.2%)	32 (76.2%)
Partial Response	17 (40.5%)		
Stable Disease	13 (31.0%)		
Disease Progression	10 (23.8%)		

**Table 3:** Safety profile (according to NCI/CTC criteria)

***Side effects***	***Number of patients with toxicity (%)***				
	
	*Grade 1*	*Grade 2*	*Grade 3*	*Grade 4*	*All grades*
*Hematological*					
Anemia	4 (9.5%)	0	0	0	4 (9.6)
Leucopenia	4 (9.5%)	2 (4.8%)	0	0	6 (14.2%)
Neutropenia	1 (2.4%)	1 (2.4%)	2 (4.8%)	0	4 (9.6%)
Thrombocytopenia	2(4.8%)	2 (4.8%)	0	0	4 (9.6%)
*Non-hematological*					
Nausea/Vomiting	5 (11.9%)	11 (26.2%)	0	0	16 (38.1%)
Mucositis	7 (16.7%)	5 (11.9%)	1 (2.4%)	0	13 (31.0%)
Neurotoxicity	8 (19.0%)	5 (11.9%)	1 (2.4%)	0	14 (33.3%)
Diarrhoea	8 (19.0%)	7 (16.6%)	2 (4.8%)	0	17 (40.4%)
Asthenia	15 (35.7%)	11 (26.2%)	3 (7.1%)	0	29 (69.0%)
Hyperbilirubinemia	1 (2.4%)	3 (7.1%)	0	0	4 (9.5%)
Anorexia	5 (11.9%)	3 (7.1%)	0	0	8 (19.0%)

**Table 4:** Toxicity-related treatment modifications

***Treatment modifications***	***No. of patients***	***%***
Any dose reduction	18	42.8%
Any cycle delay	20	47.6%
Toxicity-related withdrawals	3	7.1%

***Time to treatment modification***	***Months***	***Range***

Median time to reduction	2.0	1.0–5.3
Median time to first delay	1.3	0.5–3
Median time to toxicity-related withdrawal	3.9	1.8–6.0
